# Anti-Müllerian hormone as an ovarian reserve marker in women with the most frequent muscular dystrophies

**DOI:** 10.1097/MD.0000000000020523

**Published:** 2020-06-05

**Authors:** Olesja Parmova, Eva Vlckova, Monika Hulova, Livie Mensova, Igor Crha, Petra Stradalova, Eva Kralickova, Lenka Jurikova, Martina Podborska, Radim Mazanec, Ladislav Dusek, Jiri Jarkovsky, Josef Bednarik, Stanislav Vohanka, Iva Srotova

**Affiliations:** a Department of Neurology, University Hospital Brno; b Faculty of Medicine, Masaryk University, Brno; c European Reference Network on Rare Neuromuscular Diseases (ERN EURO-NMD), Czechia; d CEITEC – Central European Institute of Technology, Masaryk University, Brno; e Department of Neurology, Second Faculty of Medicine, Charles University, Prague and University Hospital Motol, Prague; f Department of Obstetrics and Gynaecology, University Hospital Brno; g Department of Paediatric Neurology, Masaryk University and University Hospital Brno; h Department of Clinical Biochemistry, University Hospital Brno; i Institute of Biostatistics and Analyses, Masaryk University, Brno, Czech Republic.

**Keywords:** anti-Mullerian hormone, fertility, muscular dystrophy, myotonic dystrophy, ovarian reserve

## Abstract

Some muscular dystrophies may have a negative impact on fertility. A decreased ovarian reserve is 1 of the factors assumed to be involved in fertility impairment. AMH (anti-Müllerian hormone) is currently considered the best measure of ovarian reserve.

A total of 21 females with myotonic dystrophy type 1 (MD1), 25 females with myotonic dystrophy type 2 (MD2), 12 females with facioscapulohumeral muscular dystrophy (FSHD), 12 female carriers of Duchenne muscular dystrophy mutations (cDMD) and 86 age-matched healthy controls of reproductive age (range 18 – 44 years) were included in this case control study. An enzymatically amplified 2-site immunoassay was used to measure serum AMH level.

The MD1 group shows a significant decrease of AMH values (median 0.7 ng/mL; range 0 – 4.9 ng/mL) compared with age-matched healthy controls (*P* < .01). AMH levels were similar between patients and controls in terms of females with MD2 (*P* = .98), FSHD (*P* = .55) and cDMD (*P* = .60).

This study suggests decreased ovarian reserve in women with MD1, but not in MD2, FSHD and cDMD.

## 1 Introduction

Muscular dystrophies constitute a group of inherited neuromuscular diseases with progressive muscle involvement. In the Czech population, the most common forms are Duchenne and Becker's muscular dystrophy, myotonic dystrophy, facioscapulohumeral muscular dystrophy (FSHD) and limb-girdle muscular dystrophies. In addition to symptomatic muscle involvement, other systems and organs are affected in muscular dystrophies, often including the reproductive and endocrine systems, which may have a negative impact on fertility. In men with myotonic dystrophy type 1 (MD1), reproductive problems due to a high prevalence of testicular atrophy and oligospermia or azoospermia are well-known manifestations of the disease.^[1]^ In MD1 women, fertility impairment is also considered highly probable.^[2]^ To the best knowledge of the authors, only limited and inconsistent data exist concerning the fertility of women patients with other muscular dystrophies, ie, myotonic dystrophy type 2 (MD2) or FSHD, or carriers of Duchenne muscular dystrophy mutations (cDMD).

Decreased ovarian reserve is considered 1 of the most important factors in female fertility impairment.^[3]^ Ovarian reserve may be assessed by measuring basal female sex hormones (follicle stimulating hormone [FSH], luteinizing hormone [LH], and estradiol), inhibin B, and anti-Müllerian hormone (AMH), and/or by antral follicle count. AMH is considered 1 of the most important markers of fertility in women.^[4,5]^

AMH is a glycoprotein produced by the gonads, specifically the granulosa cells of the ovarian follicles. AMH is secreted by the granulosa cells in primary, secondary, pre-antral and early antral follicles, and the number of these follicles to grow appears to be related to the size of the resting primordial follicle pool.^[6,7]^ AMH is therefore a useful endocrine marker and currently the best available measure of ovarian reserve.^[7,8]^ The primary role of AMH is to inhibit the recruitment of follicles, thus preventing premature depletion of the follicle pool.^[8]^ The dynamics of AMH levels change throughout life due the decline of the quantity and quality of the ovarian follicle pool.^[9,10]^ Different initial sizes of the follicle pool and variations in the pace of follicle pool depletion between individuals mean that major differences in AMH levels are evident in women of the same chronological age.^[11]^ Among other things AMH measurement is useful in prediction of a woman's future reproductive lifespan.^[7,8]^

The purpose of this study was to compare the ovarian reserve expressed as AMH values in women with the most frequent types of muscular dystrophy and those of healthy volunteers.

## 2 Methods

A total of 70 females with muscular dystrophies and 86 healthy controls were included in this prospective matched-case control study, carried out in the period between January 2017 and May 2018. The group of patients with muscular dystrophies consisted of 21 women with MD1, 25 women with MD2, 12 women with FSHD and 12 women with cDMD. The inclusion criteria were: age between 18 and 44 years, presence of both ovaries, and regular menstrual cycles of between 25 and 35 days. The women had not used hormones or oral contraceptive pills within the 3 months prior to participation. In the patient group, confirmed diagnosis of muscular dystrophy was also an inclusion criterion. Non-optimal compensation of thyroid disease was an exclusion criterion, but patients or controls with well-compensated thyroid disease, confirmed by normal thyroid-stimulating hormone (TSH) levels and free thyroxine (FT4) levels, were included in the study (Table 1). Patients were recruited from the national registry of muscular dystrophies and were investigated in 2 neuromuscular centres. The diagnosis of all patients recruited into the study was confirmed by means of molecular genetic testing (PCR, Southern blot, multiplex ligation-dependent probe amplification). Considering the different age distribution in particular diagnosis-related subgroups (Table 1), specific control groups were created for each patient subgroup to achieve optimal age-matching, using the propensity score method in R software with MatchIt package. Detailed data concerning these subgroups appear in Table 1. The content of the particular control subgroups was: 42 healthy controls to 21 women with MD1 (2:1 ratio), 25 healthy controls to 25 females with MD2 (1:1 ratio), 36 healthy controls to 12 females with FSHD (3:1 ratio) and 12 healthy controls to 12 carriers of Duchenne muscular dystrophy (1:1 ratio). The optimal ratio was set individually for each dataset on the basis of sufficient standardized difference between the groups analyzed after matching. All patients and controls were Caucasians. The study was approved by local ethics committee before the study began. Informed consent was obtained from all subjects.

**Table 1:**
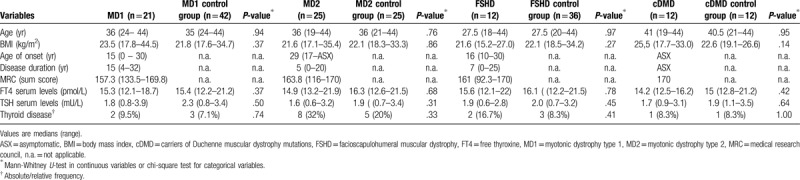
Characteristics of study participants.

AMH levels were measured in serum samples using the Beckman Coulter Access 2 immunoassay system. The Access AMH assay is a simultaneous 1-step immuno-enzymatic (“sandwich”) assay. Its design parameters include a limit of detection of ≤0.02 ng/mL (0.14 pmol/L) and a Limit of Quantitation of ≤0.08 ng/mL (0.57 pmol/L). All blood samples for the evaluation of AMH were examined in the same laboratory. The serum level of FT4 was also established in all patients and controls.

The Medical Research Council scale was used to assess the severity of muscle weakness. This scale evaluates the muscle strength of a total of 18 muscles (muscle groups), routinely using a scale ranging from 0 to 5 points for each muscle (group) for a total maximum of 170 points. Certain muscles groups were counted twice because of bilateral assessments.^[12]^

Body mass index was calculated from the weight and height established on initial examination.

The distribution of AMH, the outcomes and the covariates of interest are presented as median and range for continuous variables and the absolute/relative frequencies for categorical variables. Comparison of particular groups was performed by means of the Mann-Whitney *U* test with respect to non-normal distribution of the data. The Spearman correlation test was used for correlation analyses. The Chi-square test was employed to compare categorical data between the groups. We tested relation of each disease and AMH with age as covariate using general linear model. Statistical significance was considered as *P* < .05. Statistical analysis was performed using Statistica 12 software.

## 3 Results

Demographic and clinical data of all the patient subgroups and their controls are summarized in Table 1. No relevant differences emerged between patients with any diagnosis of interest and relevant control groups in terms of age, body mass index or history of thyroid disease. Any thyroid disease was well-compensated in all the subjects included, as documented by evaluation of FT4 serum levels, which was in normal range in all patients and controls. Patients with MD2 had shorter duration of the disease with respect to higher age of disease onset in this group, which is a feature typical of MD2 (compared with MD1). The clinical severity of the disease (expressed as medical research council sum score) showed highest variability in FSHD patients in particular (ranging from asymptomatic to moderate muscle impairment). The severity of disease in MD1 and MD2 was mostly mild, which might be anticipated with respect to the age of groups. All the carriers of Duchenne muscular dystrophy mutations were asymptomatic.

Table 2 summarizes the AMH levels in each of the patient subgroups and the comparison with relevant healthy controls. The MD1 group was the only 1 to show a significant decrease of AMH values compared with age-matched healthy controls. Apart from absolute AMH values, the number of patients/controls with AMH values outside the indicated reference range (age-related AMH nomogram)^[13]^ was also evaluated. In similar fashion to absolute AMH values, the only significant differences were found between patients with MD1 and their healthy controls, while in all the other diagnoses of interest the proportion of abnormal AMH values was similar to the age-matched control groups.

**Table 2:**
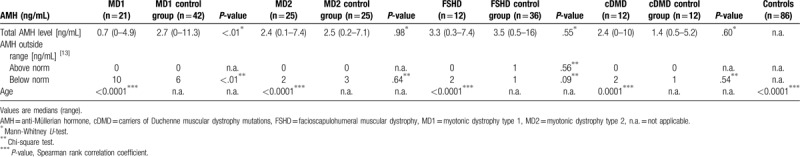
AMH levels and relationship between AMH and age.

The results of the relationship between AMH and age appear in Table 2. Significant relationships between AMH and age were disclosed in all groups of patients and in the group of healthy controls. We tested relation of disease and AMH with age as covariate and disease still plays statistically significant role for the AMH in the case of MD1 and MD2 (*P* < .05).

## 4 Discussion

Fertility disorders are a frequent and increasing problem in the general population, affecting approximately 8% to 12% of reproductive-age couples worldwide.^[14]^ Adequate ovarian reserve is 1 of the most important factors to impact upon female fertility. Several current studies have demonstrated that serum AMH concentration best represents the ovarian reserve and can thus be considered the most suitable predictor of female fertility.^[4,5]^ This is supported by the number of studies showing a strong correlation between serum AMH levels and follicle count.^[15,16]^ AMH has therefore come into widespread use in studies addressing reduced fertility in women.^[17,18]^ About 10% of healthy controls in our study had low AMH levels, which nicely corresponds to the expected prevalence of fertility problems in the general fertile population mentioned above. AMH level changes throughout life, while inter-individual variability is wide due to differences in initial follicle pool capacities and the pace of follicle pool depletion.^[7]^ Levels of AMH serum decline with increasing chronological age, and therefore age-related AMH nomograms, have been established by a number of studies.^[10,13,19]^ The current study confirmed that AMH levels are inversely correlated with age in all groups of patients and controls.

The key finding here is that a decrease of AMH concentration occurred in females with type 1 myotonic dystrophy compared with the control group and the established normal range of AMH. This finding indicates a reduced ovarian reserve and the possibility of reduced fertility potential in women with MD1. Only few reports indicate a decrease of fertility in muscular dystrophies. Almost all of them concentrate on MD1. Based on these studies, fertility in women with MD1 appears to be impaired. Female patients with MD1 have decreased ovarian sensitivity, with reduced response to ovarian stimulation and less favourable outcomes for in vitro fertilization (IVF), suggesting poor ovarian condition.^[2,20–22]^ Reduced ovarian reserve, as demonstrated by lower levels of AMH, was revealed in a recent study, to date the only 1 to determine AMH levels in MD1 females.^[2]^ This study points out that women with MD1 undergoing IVF have significantly lower AMH compared with controls (also undergoing IVF for other reasons). Our study extends these results to the general MD1 population (ie, not only those women with clinically clear fertility impairment). Furthermore, the level of AMH in our patients was very similar to that of the patients undergoing IVF in the above-mentioned study (median 0.7 ng/mL, range 0–4.9 in our study vs median 0.9 ng/mL, range 0.17–5.6 in the study cited), which clearly confirms that decreased ovarian reserve is a general feature of MD1 female patients. Decreased ovarian reserve was also observed in another study, where the day-3 FSH, estradiol, antral follicle count and number of oocytes retrieved were lower in MD1 patients, although fertilization rate and embryo quality were similar when compared with women who had undergone IVF and intracytoplasmic sperm injection in response to male infertility.^[22]^ Another authors report a higher number of poor-quality embryos and lower pregnancy rates in MD1 women.^[21]^ There are also certain studies that suggest no differences in the pregnancy rate for MD1 females.^[20,23]^ In summary, most of studies mentioned found decreased ovarian reserve in MD1 females, while others measured parameters resulted in different findings.

To the best of the authors’ knowledge, this is the first study evaluating female fertility by means of AMH concentration in patients with the other most frequent types of muscular dystrophy, ie, in MD2, FSHD, and cDMD. No significant decrease of AMH levels was found in any of these patient groups when compared with age-matched healthy controls and the established normal range of AMH in the general population. No specific data are available on female fertility in MD2 and FSHD. The carriers of X-linked diseases, including Duchenne muscular dystrophy, have been used as a control group in a study with MD1 females; normal response to ovarian stimulation was found ^21^, which is fully in the line with the observations herein.

In addition to the clear dependence of AMH level on age mentioned above, various other factors may influence AMH concentration. Undiagnosed and untreated thyroid disease can be a cause of infertility, and anovulation is typically seen in women with thyroid hypofunction.^[24]^ To exclude the influence of thyroid dysfunction on reproduction, this study measured the TSH and FT4 level in all the women involved. The concentration of TSH and FT4 in serum was normal in all patients and controls and thyroid dysfunction thus does not appear to represent a factor with potential significant influence on AMH levels.

Most studies maintain that AMH does not change significantly in the course of the menstrual cycle.^[25]^ According to the automated Access AMH assay, AMH levels appear to be relatively stable across the menstrual cycle.^[26]^ In the studies that created AMH nomograms, AMH measurement was also performed independently of the menstrual cycle.^[13,19]^ For this reason, the authors did not perform blood collections for AMH testing at any specific phase of the menstrual cycle.

Several studies described the relationship between AMH and gonadotrophins. Elevated levels of AMH suppress FSH secretion^[27]^ and also it was shown that AMH has a strong inhibitory effect on cyclic follicular recruitment in vivo by reducing the follicle sensitivity to FSH.^[28]^ But on the other hand it was demonstrated a stimulatory effect of AMH on the expression of the FSH β-chain gene in gonadotrope-derived cell lines in pituitary with an increasing the secretion of FSH without affecting LH levels.^[29]^ AMH also induces LH secretion by stimulating the activity of the hypothalamic Gonadotropin-releasing hormone (GnRH) neurons, which express AMH receptors.^[30]^ However, it has been also documented, that GnRH lowers serum AMH levels and increase the gonadotrophins (FSH and LH).^[31]^ So relationship between AMH and the hypothalamic–pituitary–gonadal axis is more complex, but shows the important role of AMH in the regulation of fertility by influencing the hypothalamic-pituitary function. Abnormalities of hypothalamic–pituitary–gonadal axis with secondary hypogonadism or combined form of primary and secondary hypogonadism have been reported in some MD1 patients.^[32]^

To the best of the authors’ knowledge, this is the first study evaluating fertility by means of AMH concentration in patients with the most frequent types of muscular dystrophy and the first study to use AMH determination for other dystrophies beyond MD1. This is also the first study to evaluate fertility by means of AMH concentration in the general population of female MD1 patients (not limited to a selected population with clear fertility impairment).

The small sample of patients constitutes a weakness of this study, although the authors investigated almost all of the women of fertile age listed in the national registry. Further studies are therefore needed to confirm or question our findings. This study presents the partial results of an extensive female fertility study of those of who suffer from muscle dystrophies, presenting the ovarian reserve as 1 of the factors that reflects women's fertility.

MD1 is a complex disorder caused by the DNA expansion in myotonic dystrophy protein kinase gene. Expression of the mutated gene gives rise to an expanded repeat RNA that is retained in the nucleus and is directly toxic to cells in affected tissue. Although the mechanism of the deleterious effects of this expansion disease on the gonads is not known, it is possible that the expanded RNA also accumulates in ovarian cells and thus may have a negative impact on the ovarian function.^[2]^ Although the MD2 is also an expansion disease like MD1 caused by the DNA expansion in cellular nucleic acid-binding protein gene, the expression of the mutated gene with expanded repeat RNA seems to be less pronounced in MD2 leading to a milder presentation of the multisystemic disorder with smaller number of affected tissues. MD2 also manifests often later at the end of the reproductive age of a woman, which could also be 1 of the reasons of normal AMH levels.

The results of this study add important considerations for MD1 patients. Women and family members should be informed about the possibility of prematurely decreasing ovarian function and high risk of impaired fertility, thus providing an opportunity for realistic reproductive planning.

## Author contributions

O. Parmova was involved in literature review, recruitment and examination of patients, data collection, data interpretation, data analysis and prepared the first draft of the manuscript. M. Hulova and L. Mensova contributed to recruitment and examination of patients and controls, data collection and data interpretation. P. Stradalova and E. Kralickova contributed to data interpretation and analysis. M. Podborska contributed to collection and interpretation of blood samples (for the evaluation of anti-Müllerian hormone). L. Jurikova contributed to recruitment of patients. L. Dusek and J. Jarkovsky contributed to interpretation of data (statistical analysis). R. Mazanec contributed to data interpretation and reviewed/edited manuscript. I. Crha contributed to study design, data interpretation and reviewed/edited manuscript. E. Vlckova, J. Bednarik and S. Vohanka contributed to study design and data interpretation, to discussion and reviewed/edited manuscript. I. Srotova assumed overall responsibility for the study, had full access to all the data in the study and takes responsibility for the integrity of the data and the accuracy of the data analysis. She contributed to study design, literature review, data collection, data interpretation, recruitment and examination of patients and controls and prepared the first draft of the manuscript. All authors approved the final manuscript.
